# The pattern of neurological diseases in elderly people in outpatient consultations in Sub-Saharan Africa

**DOI:** 10.1186/s13104-015-1116-x

**Published:** 2015-04-17

**Authors:** Kuate-Tegueu Callixte, Tchaleu Benjamin Clet, Doumbe Jacques, Yepnjio Faustin, Dartigues Jean François, Tabue-Teguo Maturin

**Affiliations:** Department of Internal Medicine and Specialties, Faculty of Medicine and Biomedical Sciences, University of Yaoundé I, Yaoundé, Cameroon; French Institute of Public Health and Medical Research, Bordeaux, F-33076 France; University Victor Segalen Bordeaux 2, Bordeaux, F-33076 France; Internal Medicine department, Douala General Hospital, Douala, Cameroon; Neurology Department, Yaoundé Central Hospital, Yaoundé, Cameroon; Faculty of Medicine and Pharmaceutical Sciences, University of Douala, Douala, Cameroon

**Keywords:** Epidemiology, Hospital prevalence, Neurological disorders, Elderly people, Urban Africa, Cameroon

## Abstract

**Background:**

Neurological diseases are frequent in older adults, affecting between 5% and 55% of people age 55 and older. They are associated with a high risk for adverse health outcomes, including mortality, disability, institutionalization and hospitalization. Little is known about the epidemiology and clinical pattern of neurological disorders of the elderly in developing countries. Although many studies have demonstrated the areas where the burden of neurological diseases lies, elderly patients in Sub-Saharan Africa have received little attention. We performed this descriptive study to understand the burden of disease faced by Cameroonian neurologists and geriatricians.

**Methods:**

The records of all out-patient neurological consultations from May 2005 to December 2011 were collected at the Clinique Bastos, the sole clinic with adult neurological consultations during the study period in Yaoundé, the political capital of Cameroon. All medical records were reviewed by a neurologist and neurological diagnoses were classified according to ICD-10.

**Results:**

Among a total of 912 patients, 187 (20.5%) were aged 60 and older. According to the ICD-10 classification, episodic and paroxysmal disorders were present in 18.7% of patients, extrapyramidal and movement disorders in 14.6%, and nerve, nerve root and plexus disorders in 13.3%. The most common neurological diseases of the elderly in this study were lumbar arthrosis (14%), dementia (Alzheimer’s type, vascular) (12.4%), Parkinson’s disease (10.2%), and polyneuropathy (9.1%).

**Conclusion:**

Degenerative diseases like dementia and Parkinson’s disease as well as strokes and headaches are frequently encountered neurological diseases in elderly patients in Sub-Saharan Africa. It is important that standard treatment regimes, often Ministry of Public Health based, are adhered to these diseases.

## Background

Neurological diseases are frequent in older adults, affecting between 5% and 55% of people aged 55 and older [1,2]. They are associated with a high risk of adverse health outcomes, including mortality, disability, falls, institutionalization and hospitalization.

Little is known about the epidemiology, natural history, clinical pattern, aetiologies and treatment status of neurological disorders in elderly people in developing countries. The overall burden of neurologic disease is increasing in the developing world [3]. In fact, most disorders of the nervous system occur in developing countries [3]. The risk of death rises rapidly among adults aged 60 and over in all regions. Globally, 60 year olds have a 55 percent chance of dying before their 70th birthday. Regional variations in the risk of death are smaller at older ages than at younger ages, ranging from around 40 percent in the developed countries of Western Europe to 60 percent in most developing regions and 70 percent in Sub-Saharan Africa [2,4].

In both high and low income WHO regions, neuropsychiatric conditions are the most important causes of disability, accounting for more than 37 percent of the years of healthy life lost as a result of disability (YLD) among adults age 15 and over. The disabling burden of neuropsychiatric conditions is almost the same for males and females, but the major contributing causes are different [2,4]. Surprisingly, almost 50 percent of the adult disease burden in low- and middle-income countries is now attributable to non-communicable disease. Population aging and changes in the distribution of risk factors have accelerated the epidemic of non-communicable disease in many developing countries [2,4].

Although we used the same cohort as in our previous study [2], the question raised here is the problem of the diagnosis and management of neurological disorders in the elderly in Cameroon and Sub-Saharan Africa. The aging population is a real public health issue not only in the West, also in Sub-Saharan Africa. It is essential to identify the incidence and prevalence of neurodegenerative diseases, in order to develop strategies for prevention and management.

One of the objectives of this study was to determine the incidence of dementias in Cameroon. These neurodegenerative diseases are certainly under-diagnosed or unrecognized in the elderly, probably because more attention is paid to infectious diseases, including malaria, HIV-AIDS and tuberculosis. The data from this cohort of patients followed up in consultations helped us to screen neurological diseases in the general population and to analyze the subgroup of elderly in order to draw the attention of governments and the medical community to the extent of this often ignored problem in Sub-Saharan Africa.

## Methods

The records of all out-patient neurological consultations from May 2005 to December 2011 were collected at the Clinique Bastos, the sole clinic with adult neurological consultations during the study period in Yaoundé, the political capital of Cameroon. For all patients aged 60 and older, the following data were extracted from the medical records: age, sex, residence, referral person or personnel, date of first consultation, main complaint for the 1^st^ consultation and diagnosis. We classified neurological diseases according to the 10^th^ revision of the international classification of diseases as follows [5,6]: (1) Inflammatory diseases of the central nervous system, (2) Systemic atrophies primarily affecting the central nervous system, (3) Extrapyramidal and movement disorders, (4) Other degenerative diseases of the nervous system, (5) Demyelinating diseases of the central nervous system, (6) Episodic and paroxysmal disorders, (7) Nerve, nerve root and plexus disorders, (8) Polyneuropathies and other disorders of the peripheral nervous system, (9) Diseases of the myoneural junction and muscles, (10) Cerebral palsy and other paralytic syndromes, (11) Other disorders of the nervous system.

The diagnoses were made clinically with laboratory and radiological confirmation. The laboratory tests depended on the suspected neurological diagnostic process and included full blood count, ESR, serum biochemistry, serological tests and microbiology. Lumbar puncture and muscle biopsy were used when indicated. The radiological tests were CT-scan (brain and spine), spine X-ray, and rarely MRI, which became available only in 2010 and was used only when indicated for financial reasons. Electrophysiological tests included electroencephalography (EEG), electroneuromyography (ENMG), and electrocardiography (ECG).

### Statistical analysis

Characteristics of individuals at baseline were presented using means and standard deviations (SD) for continuous variables, and as percentages for categorical variables. Epi Info® version 5.3.1 (CDC, Atlanta, GA, USA) was used for this analysis. P-value < 0.05 was considered statistically significant.

The present study was conducted according to the guidelines laid down in the Declaration of Helsinki. Prior to the study, local institutional ethical clearance from the National Ethics Committee was sought and obtained and an administrative authorization was obtained from the management of the Clinique Bastos. All participants gave their verbal or written consent.

## Results

### Demographics

Nine hundred and twelve patients with neurological diseases were selected from 2005 to 2011 from outpatient consultations. Out of these patients, 187 (20.5%) were age 60 and older. Baseline data included socio-demographic and lifestyle characteristics, symptoms or complaints and main chronic conditions. Table 1 shows the socio-demographic characteristics of the patients.

**Table 1:** **Socio-demographic characteristics of patients**

**Age (years)**	**Sample (N = 187): n (%)**
**60-70**	120 (64.2)
**70-80**	46 (24.6)
**80-90**	17 (9)
**>90**	4 (2.2)
**Gender, Male**	92 (49.2%)
**Place of residence**	
**Yaoundé**	132 (70.6%)
**Other nine regions of country**	44 (23.5%)
**Other countries**	7 (3.7%)
**Neighboring towns**	4 (2.1%)
**Sources of referral**	
**Specialized doctors**	111 (59.4%)
**General practitioners**	43 (23%)
**Nurses**	13 (7%)
**Alone**	19 (10.2%)
**Physiotherapist**	1 (0.5%)

The sex distribution of patients was 95 (50.8%) females and 92 (49.2%) males, giving a sex ratio (F/M) of 1.03/1. The ages of the patients ranged from 60 to 97 years with the mean of 68.83 ± 7.16 years and median of 68 years. For patients aged between 70 and 80 years, females were more numerous than males while males were more numerous for the age groups 60 to 70 years and 80 to 90 years. This difference was not statistically significant.

Out of the 187 elderly patients, 132 (70.6%) were living in Yaoundé, 4 (2.1%) came from the nearest town, 44 (23.5%) from the nine other regions of Cameroon, and 7 (3.7%) from other countries (Chad, Equatorial Guinea, Central African Republic and Gabon).

A total of 111 patients (59.4%) were referred by other specialized doctors, 43 (23%) by general practitioners, 13 (7%) by a nurse, 19 (10.2%) came alone and one (0.5%) by a physiotherapist.

### Diagnosis and presenting complaints

The 10 leading complaints are presented in Table 2. The frequency of the various neurological diseases groups according to ICD-10 classification is shown in Table 3. Episodic and paroxysmal disorders were present in 18.7% of patients, extrapyramidal and movement disorders in 14.6% and nerve, nerve root and plexus disorders in 13.3%. Out of the 187 patients, 41 (22%) finally had a non-neurological diagnosis. The frequency of the ten leading neurological diseases in outpatient consultations is presented in Table 4.

**Table 2:** **The 10-leading complaints in consultations**

**The 10-leading complaints**	**Frequency**	**Percentage**
1 Low back pain	37	19.7%
2 Tremor and movement disorders	26	13.8%
3 Pain in lower limb	20	10.6%
4 Cognitive disorders	18	9.6%
5 Headaches	17	9.1%
6 Cervical pain	13	6.9%
7 Hemiplegia	9	4.8%
8 Pain in upper limbs	9	4.8%
9 Gait disorder	8	4.3%
10 Loss of consciousness	4	2.1%

**Table 3:** **ICD-10 classification of neurological diseases in the elderly**

**ICD-10 Classification**		**Frequency**	**Percentages (%)**
Episodic and paroxysmal disorders	Headaches	14	7.5
	Epilepsy	6	3.2
	Cerebrovascular	13	7.0
	Sleep disorders	2	1
Nerve, nerve root and plexus disorders		25	13.3
Other disorders of the nervous system		11	5.8
Extrapyramidal and movement disorders		27	14.6
Polyneuropathies and other disorders of the peripheral nervous system		17	9.2
Inflammatory diseases of the central nervous system		2	1
Other degenerative diseases of the nervous system		24	12.8
Cerebral palsy and other paralytic syndromes		4	2.1
Diseases of myoneural junction and muscle		1	0.5
Systemic atrophies primarily affecting the central nervous system		0	0
Demyelinating diseases of the central nervous system		0	0

ICD: International Classification of Diseases.

**Table 4:** **The ten leading diseases in the elderly in outpatient neurological consultations in Yaoundé**

**The ten leading diseases**	**Frequency (%)**	**Male (%)**	**Female (%)**	**P-value**
**1) Lumbar arthrosis**	26 (14)	2 (7.7)	24 (92.3)	0.000*
**2) Dementia (Alzheimer = 12, vascular = 9, SNP = 2)**	23 (12.4)	16 (69.5)	7 (30.5)	0.011*
**3) Parkinson’s disease**	19 (10.2)	11 (57.9)	8 (42.1)	0.231
**4) Polyneuropathy**	17 (9.1)	8 (47)	9 (53)	0.863
**5) Intervertebral disc disorder**	15 (8)	8 (53.3)	7 (46.7)	0.500
**6) Headaches**	14 (7.5)	4 (28.5)	10 (71.5)	0.192
**7) Stroke**	13 (7)	8 (61.5)	5 (38.5)	0.213
**8) Cervical arthrosis**	12 (6.4)	3 (25)	9 (75)	0.145
**9) Depression**	7 (3.7)	3 (42.8)	4 (57.2)	0.903
**10) Essential tremor**	7 (3.7)	6 (85.7)	1 (14.3)	0.027*
**Total**	153 (81.9)	69 (45)	84 (55)	

*: statistically significant p-value; SNP: supra nuclear palsy.

## Discussion

The aim of this study was to describe the neurological diseases in an elderly Sub-Saharan African population.

The elderly over 60 years of age represented 20.5% of all outpatients’ admissions. In a study done in the Niger delta area of Nigeria, out of 1395 patients admitted in neurology in 10 years, 597 (42.8%) were age 60 years and older [7]. In a similar study in the Ivory Coast, 26.2% of admissions in neurology were 60 years and older [8]. The mean age of patients was similar in other Sub-Saharan populations but lower than that seen in developed countries. Indeed, the life expectancy of a Cameroonian citizen is 52 years compared to 77.8 years in the USA [8]. The rapidly increasing elderly population poses a major challenge for future healthcare systems. In the near future, the rising numbers of elderly patients will represent a challenge for care. The shift towards an aging society will lead to a growing number of elderly patients with neurological diseases [9].

The majority of patients in our study (82.4%) were referred by other doctors. When this study was conducted, there were no geriatricians in the country, fewer than 10 neurologists, and only four in Yaoundé. This might explain the vast majority of patients were referred by other health practitioners who found it difficult to manage older people especially when they have neurological diseases. The data from Yaoundé-Cameroon (Table 3), display the same pattern of neurological diseases as that found in the majority of tropical countries [10-12]. The predominance of episodic and paroxysmal disorders (18.7%) is similar to the findings by Anand et al (1993) in India [13], Chapp-Jumbo in Nigeria [7], Andrantseheno in Madagascar in an hospital-based study in the general population of the northwestern part of the island [14], Winkler et al in rural Tanzania [15,16], Dewhurst et al’s group of 349 people age 70 years and older in Tanzania [10,11], Walker et al in urban Tanzania [17] and Kuate et al in Cameroon [5]. However, in a Zambian tertiary care hospital study of inpatient and outpatient neurological diseases in the general population [18], infectious diseases made up the largest percentage of inpatient neurological illness while non-infectious causes were responsible for the majority of outpatient neurological cases.

The ten leading diseases in our study were lumbar arthrosis (14%), dementia (12.4%), and Parkinson’s disease (10.2%), polyneuropathy, intervertebral disc disorders, headache, stroke, cervical arthrosis, depression and essential tremor. The leading position of degenerative diseases like dementia among our hospital-based outpatient’s consultation cases confirms their importance in public health. Dementia is therefore a public health problem in Yaoundé (Cameroon) and is responsible for 12.4% of admissions in neurology outpatient’s consultations. However, this pattern is not homogeneous across the tropical countries in Africa. This result is close to that observed in the general population of an urban area of Bangui, Central African Republic (8.1%) [19] but lower than that observed in rural Tanzania (6.4%) [20], in the general population of urban Congo-Brazzaville (6.7%) [19], in a rural population aged 65 years and older in Benin (3.7%) [21] and in Nigeria (2.3%) [22]. Unlike in several Sub-Saharan African studies [19-21], males were more frequent than females in our dementia cases. This difference could be due to the small sample size.

A study by Chapp-Jumbo in Nigeria reported stroke in 61.6% of cases followed by meningitis and encephalitis in 13.4% [7]. Another study in Nigeria by Osuntokun [6] reported infections of the nervous system to be the commonest neurological problem, followed by vascular disease and epilepsy. In Ivory Coast, stroke was reported in 42.18% followed by cerebral toxoplasmosis (17.9%) and meningo-encephalitis in 11.9% in a hospital-based study [23]. In Ethiopia [24], cerebrovascular disease (45%) was the most common neurological pathology seen, followed by bacterial meningitis (12%). In Nairobi-Kenya [25], the 3 commonest diseases were meningitis (23.1%), epilepsy (16.6%) and cerebrovascular diseases (15.0%). In a rural area of Tanzania [11], the age-adjusted prevalence per 1000 of the most common neurological disorders in older people were tremor (48.2), headache (41.8), stroke (23.0) and polyneuropathy (18.6). Indian studies reported a high prevalence of epilepsy in 20.6% [13] while in rural Tanzania, the prevalence of epilepsy was 2.91/1000 adults [26] and 4.9% in a rural area of Bilomo in Cameroon [27]. Other studies in Sub-Saharan Africa have reported crude yearly stroke incidence rates of 94.5-107.9/100, 000 [17], 20/100,000 for Parkinson disease [28], 41/100,000 for essential tremor [29], 4.3% for 1-year prevalence of migraine headache [16], and 0.1% for hospital-based prevalence of Parkinsonism [30]. The classification of neurological diseases in the elderly in urban Africa is important in terms of diagnostic approach, therapy and prognosis. Diagnoses relating to the ten most frequent diseases (see Table 4) should be made as early as possible.

Concerning stroke (7% of the patients in our study), its incidence is probably underestimated owing to outpatient recruitment and loss to follow up. Another reason may be the high prevalence of high blood pressure in this urban setting, which is usually underdiagnosed and is responsible for stroke at a younger age [31]. Reported stroke prevalence varies according to study setting and design. In the study in elderly people in Tanzania, stroke prevalence was 23.0 per 1000 [11]. In Sub-Saharan Africa, the prevalence of stroke tends to be low owing to high mortality [17,32,33].

Neurodegenerative and cerebrovascular diseases are very frequent in the elderly. Their prevalence increases from age 55–65 years to age 90 years and ranges from less than 1% to over 40% for dementia, from less than 0.5% to more than 4% for Parkinson’s disease [34], and from approximately 1% to nearly 10% for stroke. Nearly 20% of elderly people have at least one silent brain infarct and therefore run a nearly fourfold increased risk of clinical stroke and a more than two-fold risk of dementia including Alzheimer’s disease [35].

In our study, non-infectious neurological diseases were more common than infectious diseases. This is consistent with previous reports showing that the incidence of non-communicable neurodegenerative diseases will increase in the developing world and that the death rates from non-communicable diseases are higher in the developing world than in developed regions such as the USA and Europe [4]. Similarly, although HIV infection represents much of the neurologic morbidity and mortality in Sub-Saharan Africa, the majority of neurological diseases in elderly patients are not HIV-related [12,15]. In terms of resource allocation, the high burden of neurological disease will require specialists capable of differentiating between all possible illnesses to older people as well as the nervous system. High-quality neurologic and geriatric training is essential to tackle this huge and increasing number of patients [36].

Post-graduate training is needed to improve the diagnosis and management of the most frequent forms like arthrosis, the dementias and Parkinson’s diseases. Drugs availability and cost must also be taken into account owing to the need for long term treatment. Indeed, patient compliance depends partly on these extra-medical factors.

An important and unexpected lesson from the Rotterdam Study [1,37,38] is the large potential for the prevention of diseases in elderly people, or at least for the postponement of their clinical manifestations. There is considerable scope for acting upon vascular conditions, oxidative stress, and inflammatory factors. The vascular factors that could be targeted to prevent neurological diseases in elderly people, especially dementia, include the classic cardiovascular risk factors such as high blood pressure, high cholesterol level, and smoking, atherosclerosis and conditions that contribute to it such as diabetes mellitus and atrial fibrillation [38].

The most obvious limitation of this study is the hospital-based design. Viral studies, polymerase chain reaction (PCR), histochemistry and other high technology neurologic investigative tools were not used for the diagnoses of these patients because these facilities were not available. Neurological disease diagnosed in an urban setting does not necessarily represent its prevalence in the community or the whole country. However, it does represent the disease seen by neurologists in the country because they all work in the major cities.

The risk factors for these neurological diseases clearly need to be studied. The challenge is to establish the causes of diseases in the elderly population in Sub-Saharan Africa by performing a prospective follow-up study. The two basic measures of disease frequency are prevalence and incidence. Prevalence describes the number of cases found in a cross-sectional survey while incidence describes the number of new cases that develop within a period of observation that takes into account the time that has lapsed from baseline and the number of people under observation during that time (person-years). While prevalence data are particularly relevant for health service planning, incidence data provide the basis for furthering our understanding of the etiology of these diseases [39].

## Conclusions

The neurological diseases diagnosed in the elderly population in Yaoundé-Cameroon are globally similar to those in the majority of tropical countries. Degenerative diseases like dementias and Parkinson’s disease as well as stroke, headache and intervertebral disc disorders are the most frequent. The top ten diseases should be diagnosed as early as possible as often there are crucial therapeutic implications and preventive measures to be taken. Standard treatment regimes, often Ministry of Public Health-based, should be adapted to these diseases. In addition, the national authorities in Cameroon should re-inforce lectures and/or training in geriatric medicine.
